# Dental Caries Is Associated with Multidimensional Poverty: Evidence from Colombia

**DOI:** 10.3390/healthcare14050590

**Published:** 2026-02-27

**Authors:** Mauricio Alberto Cortes-Cely, Luis Jorge Hernandez-Florez, Angelica Castro-Rios, Monica Pinilla-Roncancio, S. Aida Borges-Yañez

**Affiliations:** 1 Programa de Maestría y Doctorado en Ciencias Médicas, Odontológicas y de la Salud, Universidad Nacional Autónoma de México, Ciudad de México CP 04510, Mexico; ma.cortes140@uniandes.edu.co; 2 Facultad de Medicina, Universidad de los Andes, Bogotá D.C. CP 111711, Colombia; luishern@uniandes.edu.co; 3 Centro de los Objetivos de Desarrollo Sostenible para América Latina y el Caribe, Universidad de los Andes, Bogotá D.C. CP 111711, Colombia; 4 Unidad de Investigación en Epidemiología Clínica, Hospital de Pediatría, Instituto Mexicano del Seguro Social, Centro Médico Nacional Siglo XXI, Ciudad de México CP 06720, Mexico; angelica.castror@imss.gob.mx; 5 División de Estudios de Posgrado e Investigación, Facultad de Odontología, Universidad Nacional Autónoma de México, Ciudad de México CP 04510, Mexico

**Keywords:** dental caries, oral health, social determinants of health, poverty

## Abstract

Objective: The aim of this study was to investigate the association between dental caries and multidimensional poverty in Colombia using data from the National Oral Health Survey (ENSAB IV, Spanish acronym). Methods: A cross-sectional analytical study was conducted using data from 20,534 individuals in six regions of the country. Dental caries was assessed using the ICDAS system, and multidimensional poverty was measured using a proxy Multidimensional Poverty Index (MPI) adapted from the method adjusted for Colombia. Descriptive analyses and bivariate comparisons were carried out, and Poisson regression models adjusted for sociodemographic variables were applied. Results: Households containing at least one member with caries had a higher incidence (59.9%) and intensity (46.7%) of multidimensional poverty compared to those without caries (52.6% and 45.6%, respectively). Significant associations were identified between caries and deprivation in education (low educational attainment: RR = 1.27), child labor (RR = 1.16), unemployment (RR = 1.04), lack of health insurance (RR = 1.09), and inadequate housing conditions (RR = 1.19). The model that analyzed the presence of caries in a household and multidimensional poverty, when controlled for housing conditions, confirmed a positive association between the MPI and the presence of caries (IRR = 1.08; 95% CI: 1.050–1.107; *p*-value < 0.001). A female head of household and rural residence were also identified as variables associated with the presence of caries in a household. Conclusions: The presence of a household member with dental caries is significantly associated with multidimensional poverty in Colombia. This study highlights the need to consider oral health as a sensitive indicator of structural inequality and proposes its inclusion in social progress metrics. The findings support the design of comprehensive public health strategies that address the social determinants of oral health, especially in vulnerable populations.

## 1. Introduction

Dental caries is one of the most prevalent chronic diseases worldwide and constitutes a major public health problem in multiple countries [1]. Several studies have indicated a direct relationship between socioeconomic conditions and oral health, identifying monetary poverty as a key determinant in the onset and progression of dental caries [2]. This relationship becomes evident through multiple mechanisms, including limited access to products and services in oral health, and the adoption of less healthy eating habits, often associated with contexts of scarce economic resources [3]. However, these financial factors do not act in isolation. Non-economic social and commercial determinants, such as caregivers’ education level, cultural practices related to oral hygiene, and the availability of health services, are significantly associated with the prevalence of dental caries, especially in children [4,5]. These determinants act in a complex and interrelated manner, requiring interventions and in-depth analysis that goes beyond the financial dimension.

The scale of this challenge is clearly reflected in the Colombian context. The Fourth National Oral Health Survey (ENSAB IV), which aimed to describe the oral health profile of the Colombian population, found that the presence of caries—defined as the percentage of people who show evidence of having experienced any of the stages or sequelae of caries—increases significantly with age. Specifically, its prevalence rises from 6.02% at one year of age to 62.10% at five years, eventually reaching 96.26% in older adults [6]. To understand these figures within a broader social framework, it is essential to consider the methodology proposed by Alkire and Foster for measuring multidimensional poverty. This approach understands poverty as a condition where people experience simultaneous deprivations across multiple essential domains, such as health, education, and standard of living. By employing a dual-cutoff approach, the Alkire–Foster (AF) method measures both the incidence (H) and the intensity (A) of the average number of weighted deprivations that people living in poverty face, a model now used in more than 40 countries to compute the Multidimensional Poverty Index (MPI) [7,8].

In 2010, the Colombian government adapted this method by defining a measure composed of five dimensions and 15 indicators. Under this framework, a household is considered poor if it scores 33% or higher, reflecting deprivation in at least five weighted indicators. Because the household is the unit of identification, if one member is deprived in an indicator, all members are considered deprived [9]. This distinction is crucial because, as several authors have noted, the impact of poverty on health goes far beyond a lack of income [3,10,11]. A person living in poverty may face simultaneous disadvantages, such as low educational attainment, unemployment, food insecurity, poor housing conditions, and limited access to healthcare services, which increases their biological, social, and structural vulnerability. In this sense, poverty acts as a fundamental social determinant of health that conditions both exposure to risk and the capacity to respond to and recover from illness [8,12].

Furthermore, the household serves as the primary space where these structural determinants materialize. In the case of oral health, outcomes like dental caries are deeply influenced by shared conditions, including dietary patterns, access to fluoridated water, and the availability of economic resources for hygiene products. Consequently, deprivation in one member’s oral health serves as a proxy for the household’s collective vulnerability to structural poverty. Despite this clear connection, few studies in the field of dentistry have explored the specific association between multidimensional poverty and oral health [13,14,15]. Unlike previous self-reported research, the present study is based on clinical and social data collected at the national level, providing greater methodological rigor. Therefore, the purpose of this study is to establish, for the first time, the association between dental caries and multidimensional poverty in the Colombian population.

## 2. Materials and Methods

This is a cross-sectional analytical study.

### 2.1. Data and Sources of Information

Microdata from the latest version of the Fourth National Oral Health Survey (ENSAB IV) were used. This survey was conducted in Colombia between 2013 and 2014 by the Colombian Ministry of Health and Social Protection; details regarding the data collection instruments and variables are available in the official ENSAB IV report [6,16,17].

The sample for this study was composed of members of the Colombian population aged 1, 3, 5, 12, 15, and 18 years, which adheres to the World Health Organization (WHO) guidelines for oral health surveys [18]; pregnant women between 20 and 49 years of age; and adults (excluding pregnant women) between the ages of 20 and 79, all living in private homes in any of the 16 subregions that make up the country’s Atlantic, Eastern, Central, Pacific, Bogotá, and Orinoquía/Amazonía regions. People living temporarily or permanently in collective lodgings such as hospitals, military barracks, prisons, convents, welfare facilities, and similar facilities were excluded.

For the identification and selection of the Primary Sampling Units (PSUs), the authors considered the political and administrative divisions of the country and the population projections from 2006 to 2020, both national and municipal, stratified by sex and age group, as projected by the National Administrative Department of Statistics [19].

The sample included a total of 20,534 participants, with 31.4% (n = 6446) aged 1–3 and 5 years old; 29.4% (n = 6046) aged 12, 15, or 18; 13.9% (n = 2863) aged 20 to 34; 8.2% (n = 1689) aged 35–44; 11.2% (n = 2301) aged 45–64; and 5.8% (n = 1189) aged 65–79. This population had a distribution of 48.7% men and 51.3% women.

The group of pregnant women included 1050 participants, with 10.0% (n = 105) aged 12–18; 80.7% (n = 847) aged 20–34; 9.1% (n = 96) aged 35–44; and 0.2% (n = 2) aged 45–64.

All individuals underwent a clinical oral examination to assess the following conditions: cleft lip and palate, potentially malignant lesions of the oral mucosa, dental caries, occlusal abnormalities, dental fluorosis, edentulism, use of or need for dental prostheses, periodontal disease, opacity, trauma, and erosion. The survey had a notable level of disaggregation for accurate estimates in terms of region, subregion, area, age or age group, sex, and registration status when compared with the General Social Security System for Health (SGSSS) levels [16].

In terms of reference periods, the time frames explored for the different factors were as follows: for income, one month; for lifestyles and oral health, 12 months and throughout an individual’s life; and for lifestyles and behavior, one year, the last six months, and the last week.

Data collection was conducted between 2013 and 2014, and the sampling design was based on the official population projections for the period 2006–2020 provided by the National Administrative Department of Statistics (DANE). These projections were used solely to define the sample size and select the PSU. Finally, expansion factors were calibrated to December 2013, incorporating official adjustments for population density, socioeconomic strata, and public utilities coverage across municipalities [17].

### 2.2. Variables

In Colombia, since 2010, the DANE has calculated the national MPI annually using the National Quality of Life Survey (ENCV), using a methodology adjusted to the country’s conditions [20].

The MPI in Colombia is composed of five dimensions and fifteen variables: (A) educational conditions (1. illiteracy and 2. low educational attainment); (B) conditions of children and youth (3. school non-attendance, 4. school lag, 5. barriers to access to early childhood care services, and 6. child labor); (C) work (7. informal work and 8. long-term unemployment); (D) health (9. lack of health insurance and 10. barriers to accessing health services when needed); and (E) access to public utilities and housing conditions (11. lack of access to improved water sources, 12. inadequate excreta disposal, 13. inadequate flooring materials, 14. inadequate wall materials, and 15. critical overcrowding) [21].

**Figure 1:**
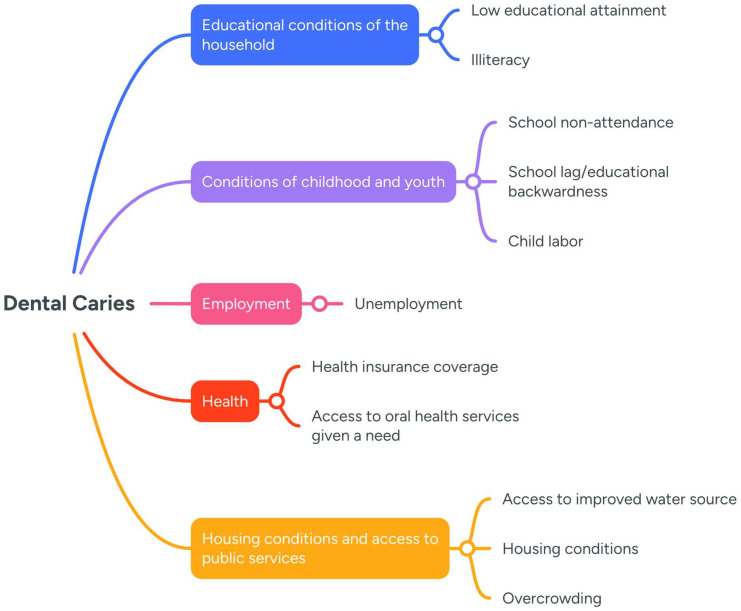
A conceptual model of the relationship between dental caries and the dimensions and variables of the Multidimensional Poverty Index in Colombia.

In this study, a proxy MPI was designed according to the data available in ENSAB IV (shown in Figure 1), which, due to data limitations, excludes some indicators of the official national MPI (barriers to access to early childhood care services, informal work, inadequate excreta disposal, inadequate flooring material, and inadequate wall material).

Although we aimed to compute as many indicators included in the national MPI for Colombia as possible, we did not aim to obtain a comparable result to the one produced by the national MPI using the ENCV for Colombia. Instead, we aimed to understand how the concept of multidimensional poverty related to dental caries using a proxy for a defined and accepted measure of poverty in the country. A list of the variables included in each dimension of the proxy model is presented in Table A1.

The methodology employed a set of basic dimensions of poverty, such as education, health, housing conditions, work, and access to public services. Within each dimension, specific indicators were defined, and a deprivation threshold was established for each one, allowing us to establish whether a person or household was deprived in that dimension [22].

Next, a cumulative assessment was conducted, whereby the deprivation affecting each household was added up. To determine whether a household is poor in multidimensional terms, a poverty threshold (33%) was used; if a household was deprived in one-third or more of the weighted indicators, they were classified as multidimensionally poor.

The MPI combined two main components: incidence (H), which represented the percentage of people considered multidimensionally poor, and intensity (A), which expressed the average deprivation faced by these people. Consistent with the conceptual framework described previously, the MPI was calculated using the AF method represented by the following formula [23]:

MPI = H × A(1)

The MPI ranges from 0 (no poverty) to 1 (all households are poor and deprived in all indicators).

### 2.3. Dental Caries

Caries was defined as a participant in whom one, two or more teeth had an ICDAS code equal to or greater than 3 (untreated caries). For household classification, when at least one member of the household had caries, the entire household was considered deprived due to caries.

To ensure methodological consistency with the Alkire–Foster method used for the Colombian MPI, dental caries was operationalized as household-level deprivation. Following the ‘all-or-nothing’ principle of MPI indicators, a household was categorized as having the outcome (1) if at least one examined member presented with dental caries, and (0) otherwise. This approach allows for a direct comparison between multidimensional poverty levels and the presence of oral disease within the same social unit.

### 2.4. Analytical Strategy

Deprivations were estimated at the household level using data collected by ENSAB IV for the 11 indicators of the proxy MPI (Figure 1). Inferential statistical tests were applied to evaluate differences between households with and without caries. In the case of categorical variables, a chi-square test was conducted. For continuous variables, Student’s *t*-test for independent samples was performed.

Subsequently, a disaggregated analysis was conducted for each of the multidimensional poverty indicators for households with and without members with dental caries. The incidence, intensity, MPI, and uncensored (percentage of households deprived in each indicator) and censored headcount ratios (percentage of households deprived in an indicator and multidimensionally poor) of households with and without members with dental caries were compared. All analyses were adjusted for the expansion factors provided in ENSAB IV, and 95% confidence intervals were calculated.

Using this information, an analysis of the distribution of deprivation and of the association between the MPI and the presence of dental caries was performed. A Poisson regression model was constructed using the incidence of multidimensional poverty as a dependent variable, which was adjusted by aspects associated with household characteristics, including the presence of at least one member with caries; the sex, age, education, and ethnicity of the head of household; the area of residence; socioeconomic stratum; and region. All analyses were performed using Stata SE 18.5 software.

### 2.5. Unit of Analysis

The unit of analysis for this study was household. All regression models were estimated using only household characteristics such as age, sex, and education of the head of the household. We analyzed the information of the MPI from the perspective of individuals living in multidimensionally poor households, given that in most cases, poor households are larger than non-poor households, and the incidence change in the information was read at the individual or household level.

### 2.6. Ethical Considerations

This study was conducted in full accordance with the Declaration of Helsinki. The research protocol was reviewed and approved by the Ethics Committee of the School of Dentistry, Universidad Nacional Autónoma de México (Approval No. CIE/0108/05/2019), and the School of Medicine, Universidad de los Andes (Approval No. 20190219). The data provided by the Ministry of Health were anonymized, ensuring the confidentiality of all participants.

## 3. Results

### 3.1. Descriptive Analysis

Table 1 shows the characteristics of individuals according to the presence or absence of untreated dental caries among different sociodemographic groups. First, women reported a higher prevalence of caries in one (17.7%) and two or more teeth (16.2%) compared to men (15.8% and 15.8%) (*p*-value = 0.006). Additionally, individuals with caries in two or more teeth (31.4 ± 20 years) tended to be older than those without caries (18.7 ± 20.6 years).

Regarding type of health insurance within the general social security healthcare system, it was observed that people who do not have health insurance as part of the subsidized system have the highest prevalence of caries (37.7% and 36.6%, respectively; *p*-value = 0.000). Regarding ethnic group, the prevalence of caries in two or more teeth was observed in 23.2% of indigenous people, 18.9% of Afro-Colombians, and 16.4% of mixed-ethnicity individuals (*p*-value = 0.000). Among people who consider themselves forcibly displaced, 20% had caries in two or more teeth (*p*-value = 0.000)

The analysis of the characteristics of households with and without caries revealed that the highest percentage of households containing one or more members with caries was located in scattered rural areas (51.2%), followed by populated areas (47.8%), and finally, municipal capitals (39.7%) (*p*-value = 0.000). In addition, forty-one percent of female-headed households and 41.7% of male-headed households had a member with caries (*p*-value = 0.11). The average age of the head of household was 45.3 ± 14.1 years in households with at least one member with caries, compared to 45.9 ± 14.3 years in households without members with caries (*p*-value = 0.000). In 73.4% of households where the head of household had a postgraduate degree, none of the members had caries. In contrast, in households where the head of household had no formal education, the percentage was 51.3% (*p*-value = 0.000). Finally, the highest percentage of households with caries was found in the Atlantic region (50.7%). (Table 1).

### 3.2. Multidimensional Poverty and Dental Caries

Table 2 shows the findings of the MPI analysis disaggregated by 11 indicators, estimating the probability of a household having at least one member with caries among households classified as multidimensionally poor (censored headcount ratios). The adjusted model shows that multidimensionally poor households with low educational attainment (RR = 1.24), child labor (RR = 1.10), long-term unemployment (RR = 1.03), no health insurance (RR = 1.08), poor housing conditions (RR = 1.12), and overcrowding (RR = 1.05) are the most likely to have a member with dental caries. Having access to healthcare services is associated with a 16% lower probability of having caries (RR = 0.84), whereas variables such as illiteracy (RR = 1.00, *p*-value = 0.83), school non-attendance (RR = 1.03, *p*-value = 0.35), and lack of access to improved water sources (RR = 1.02, *p*-value = 0.48) showed no statistically significant association with dental caries.

Table 3 presents the findings of the analysis of the MPI disaggregated at the national level and according to the presence (or absence) of dental caries in household members. Three key indicators are reported: incidence (percentage of households in multidimensional poverty), intensity (percentage of deprivations among poor households), and the composite value of the MPI. At the national level, 55.7% of the population is in a situation of multidimensional poverty, with an average intensity of 46.1%, which translates into an MPI of 0.256. When disaggregated by oral health status, we observed that households with dental caries have a significantly higher incidence of multidimensional poverty compared to households without caries (59.9 vs. 52.6%). Also, they present higher intensity (46.7 vs. 45.6%) and a higher MPI. Therefore, a larger proportion of households with caries are poor and, on average, face more deprivations, and their levels of multidimensional poverty are higher compared to households without caries.

**Figure 2:**
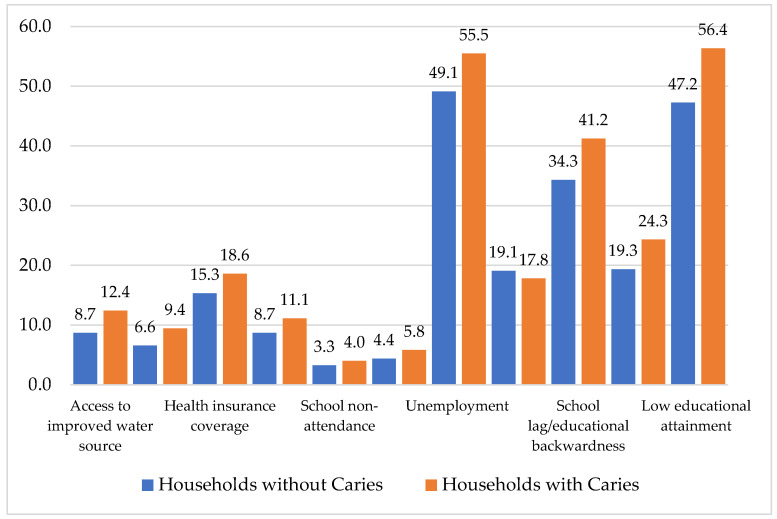
Percentage of people who are multidimensionally poor and deprived in each indicator by household with and without dental caries.

Figure 2 illustrates the censored headcount ratios, or the percentage of people deprived in each indicator and classed as multidimensionally poor. The results reveal that households with at least one member with caries present higher percentage of deprivation in all indicators (except access to dental services) and are multidimensionally poor. The largest difference is observed in low education attainment, where households with members with caries present 9.1 percentage points higher deprivation and are poor in comparison to households without caries. The second indicator is school lag, with a difference of 6.9 percentage points. In the case of access to odonatological services, 17.9% of households with caries are deprived of this indicator and multidimensionally poor in comparison with 19.1% of households without caries. The indicators with a lower percentage of people who are deprived and multidimensionally poor are school non-attendance and child labor, with 4.0 and 5.8% of households with caries deprived and multidimensionally poor, respectively.

**Figure 3:**
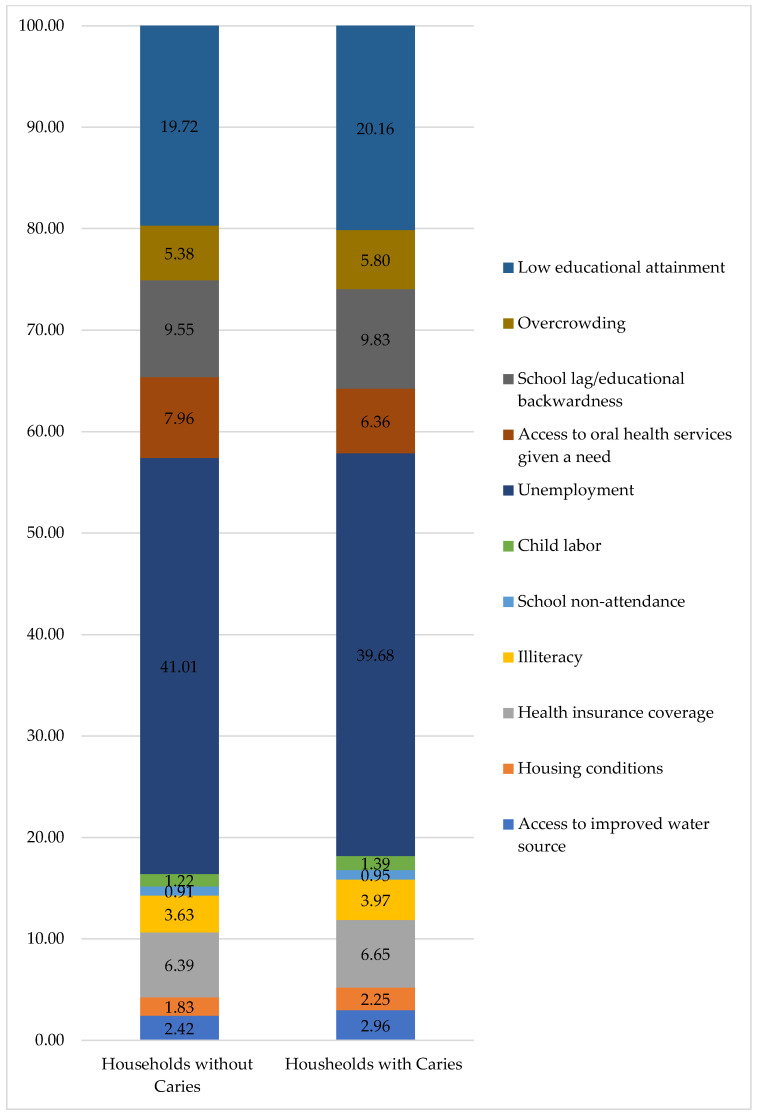
Percentages of contribution of each indicator to MPI for households with and without caries.

Figure 3 shows the percentage contribution of the eleven indicators to the MPI in households with and without caries. The group with caries has a higher relative contribution of indicators such as school lag, low educational level, overcrowding, child labor, and limited access to oral healthcare services. In contrast, the group without caries shows a more balanced distribution among the indicators, with less relative weight given to the most severe deprivations.

### 3.3. Presence of Caries According to Multidimensional Poverty and Other Demographic Variables

Table 4 presents the findings of the Poisson regression model evaluating the association between the presence of caries in a household, multidimensional poverty, and factors such as the sex, age, educational level, and ethnicity of the head of household; area of residence; socioeconomic status; and region.

The MPI is positively [14] associated with the presence of caries in a household (IRR = 1.078; 95% CI: 1.050–1.107; *p*-value < 0.001), indicating that the greater the multidimensional poverty, the higher the prevalence of caries. It was observed that female-headed households have a significantly higher likelihood of presenting caries (IRR = 1.03; 95% CI: 1.00–1.06; *p*-value = 0.030) and that the older the head of the household, the lower the probability of living in a household with at least one member with dental caries (IRR = 0.997; 95% CI: 0.996–0.998; *p*-value < 0.001).

In terms of the head of the household’s educational level, compared to having no formal education, having a secondary education is associated with a lower likelihood of presenting caries (IRR = 0.926; 95% CI: 0.873–0.982; *p*-value = 0.010), as is having technical or higher education (IRR = 0.795; 95% CI: 0.744–0.849; *p*-value < 0.001).

Regarding the ethnicity of the head of household, compared to indigenous ethnic groups, the mixed-ethnicity (IRR = 0.931; 95% CI: 0.885–0.980; *p*-value = 0.006), white (IRR = 0.876; 95% CI: 0.830–0.924; *p*-value < 0.001), and other (IRR = 0.817; 95% CI: 0.772–0.864; *p*-value < 0.001) groups exhibit a lower probability of having caries. Compared to urban areas, living in a densely populated area increases the probability of presenting caries (IRR = 1.112; 95% CI: 1.064–1.163; *p*-value < 0.001), and people in rural areas have a higher probability of living in a household with caries in comparison to those living in urban areas (IRR = 1.167; 95% CI: 1.122–1.214; *p*-value < 0.001).

Using stratum 1 as a reference, this model indicates that individuals living in higher strata have a significantly lower probability of having caries. In particular, stratum 4 shows the greatest reduction in the probability of caries (IRR = 0.804; 95% CI: 0.727–0.888; *p*-value < 0.001), followed by stratum 5 (IRR = 0.841; 95% CI: 0.804–0.879; *p*-value < 0.001) and 6 (IRR = 0.860; 95% CI: 0.826–0.896; *p*-value < 0.001).

Finally, the analysis by geographic region revealed significant differences in the probability of households having dental caries. Taking the Atlantic region as a reference, households in all other regions had a lower probability of caries.

## 4. Discussion

The purpose of this study is to identify whether the prevalence of dental caries in the Colombian population is associated with multidimensional poverty and to identify which indicators have the largest contribution to the presence of caries in a household. We found that households with higher levels of deprivation in school attainment, school lag, long-term unemployment, child labor, health insurance, overcrowding, and housing conditions were most associated with dental caries. Similarly, it was found that the incidence, intensity, and MPI were higher in households with at least one person with dental caries.

Furthermore, when multidimensional poverty was analyzed as a variable associated with living in a household with caries, it was found to exhibit a significant positive association, thus suggesting that living in multidimensional poverty increases the chances of caries compared to other household conditions. Other variables associated with living in a household with dental caries were a female head of household, being indigenous, living in a rural area, having a low socioeconomic status, and living in the Atlantic region.

The higher prevalence of caries in households with educational deprivation, school lag, and poor housing conditions suggests that social vulnerability extends beyond income to interrelated factors affecting the ability to prevent and treat caries [2,14]. These deprivations impact both general and oral health [24], with limited access to goods, services, education, and employment acting synergistically to deteriorate oral health status [25].

Specifically, a lack of education may lead to lower oral health literacy and awareness regarding oral disease prevention. Furthermore, even when awareness exists, financial constraints and a lack of medical insurance often restrict access to hygiene products and professional dental services. Finally, inadequate sanitary conditions and a lack of potable water access have been linked to increased consumption of sugary beverages as substitutes for water [26].

This is the first study to explore the association between multidimensional poverty and dental caries at the household level. The findings of this study reinforce the hypothesis that structural and social conditions contribute significantly to oral health, consistently identifying a higher prevalence of adverse socioeconomic conditions in the group with caries compared to the group without caries. Associations with factors such as limited access to oral health services, overcrowding, unemployment, child labor, and low educational attainment show significant differences, suggesting a significant link between the presence of caries and multiple dimensions of social vulnerability. These findings reinforce the understanding that dental caries is not only a public health problem but also a sensitive indicator of structural inequality. As pointed out by Listl and Bärnighausen [15], it is advisable that oral health be used as an indicator of social progress. The information obtained in this research supports this proposal, as it was identified that dental caries is positively associated with the components of the MPI and with the index as a whole.

Similarly, through information obtained from analyses such as this one, it is possible to address, from an oral health perspective, Goal 3 of the Sustainable Development Agenda: “Ensure healthy lives and promote well-being for all at all ages” [27].

These findings coincide with those of a study conducted in Colombia, which identified that consultations for hypertension and dental caries prevailed in circumstances of low human development indices (HDIs), high rates of multidimensional poverty, and high inequality (Gini) [28].

Similarly, the findings of this study are consistent with those of previous research that has documented the relationship between socioeconomic inequalities and oral diseases in low- and middle-income countries (LMICs), where lack of access to dental services and limited availability of resources for oral hygiene are recurring factors [2,10]. However, our analysis provides a novel approach by incorporating the Multidimensional Poverty Index (MPI) as a comprehensive measure, which allows us to capture the complexity of simultaneous deprivations and their cumulative impact on oral health.

The use of the Alkire–Foster method [8] made it possible to identify how these deprivations interact and accumulate, creating structural conditions that boost the onset and severity of dental caries. In this regard, the findings obtained in this study are in line with the “social determinants of health” approach, which argues that people’s health is strongly influenced by the social, economic, and environmental conditions in which they live [25,29,30]. Thus, households that simultaneously face low educational levels, a lack of access to health services, precarious housing, and other deprivations are more likely to have poorer oral health indicators, specifically dental caries.

Accordingly, the fact that commercial determinants of health are primarily negatively correlated with public health, including oral public health, in low- and middle-income countries [31] should be taken into consideration. A clear example is the promotion and encouragement of the consumption of sugary drinks, which, in addition to their impact on the incidence of diabetes and obesity, also influence the incidence of caries; this is reflected in the observation that consumption of sugary drinks is higher in people with low educational levels and low incomes, and who reside in places with inadequate public services, such as water and drainage, key elements of multidimensional poverty [14,26,32,33,34]. Similarly, in Colombia, it has been found that carbohydrate consumption is higher among people with lower socioeconomic status and lower levels of education [35].

One of the most relevant trends observed in this study is the higher association of dental caries in female-headed households. This finding can be interpreted considering the feminization of poverty and the multiple responsibilities faced by many female heads of household [36], which restricts their access to economic resources and the time they are able to dedicate to self-care and healthcare. In addition, in households with caries, the head of household generally has a lower level of education, which could be associated with restricted access to information on prevention, healthy habits, and use of dental services.

The variable “Access to oral healthcare when needed” emerges as a key protective factor, significantly reducing the incidence of caries when households have timely access to dental services. This result underscores the importance of ensuring the availability and accessibility of dental care through public policies aimed at reducing health inequalities. In this regard, the inclusion of oral health indicators in social welfare metrics could help to highlight these inequalities and guide more comprehensive interventions.

The MPI value observed in households containing members with caries (0.280) compared to those without caries (0.240) reflects a substantial difference that is attributed not only to individual factors, but also to structural determinants such as a female head of household, rural residence, and low educational attainment of the head of household—variables that remained significant in the adjusted model. These findings are consistent with studies identifying an interaction between gender, education, and geographic location as critical determinants of health [10].

Another relevant observation is that the value of the national MPI estimated using the proximal model (0.256) is equivalent to the official MPI reported by DANE for the same period (0.248), which validates the methodological approach used in this study and reinforces the reliability of the findings. This agreement suggests that, even with limitation concerning the availability of variables, ENSAB IV allows for a robust approximation for assessing multidimensional poverty. The literature supports the practice of validating alternative MPI estimates against official figures, as indicated by Sánchez Torres [37], who demonstrates that robust methodological approaches can provide findings consistent with official measurements, even under information constraints.

From a public health perspective, the findings of this study suggest that dental caries can be considered a sensitive indicator of social inequality, highlighting the need for intersectoral strategies that integrate education, housing, and access to health services. Furthermore, the evidence obtained in this study, based on national clinical and social data, contributes to closing the knowledge gap regarding the relationship between multidimensional poverty and oral health in Latin American contexts, providing input for the formulation of equity-oriented policies and encouraging similar analyses of the pattern of caries prevalence in the Global South.

Although the effect sizes for individual poverty indicators were modest, the consistency of the associations across different dimensions of the MPI supports the hypothesis of a structural gradient in oral health. No single socioeconomic factor acts as a determinant in isolation; rather, it is the accumulation of these disadvantages that creates susceptibility to disease.

It should be noted that this study has some limitations that must be considered when interpreting the findings. First, the cross-sectional design prevents the establishment of causal relationships between multidimensional poverty and dental caries, limiting temporal inference. Second, a proxy MPI was employed rather than the official national index, as the ENSAB IV survey was not originally designed for poverty measurement. Consequently, certain nuances of labor informality or specific housing deficiencies might be underrepresented. However, the construct validity remains sufficiently robust for the purpose of this analysis, as the proxy index still captures severe and simultaneous deprivations across the five dimensions, offering a reliable approximation of households’ structural vulnerability. Furthermore, the distribution of dental caries by age was not considered.

It is recommended that longitudinal studies be conducted to evaluate the causal relationship between multidimensional poverty and oral health. In addition, researchers should explore mediating mechanisms, including cultural and behavioral factors, that would enable decision-makers to develop public policies that support the generation of more effective strategies.

Finally, the relevance of these findings extends to current public health planning. Although the data correspond to the 2013–2014 period, the structural nature of the association between multidimensional poverty and dental caries suggests that these patterns are persistent. Furthermore, as Colombia anticipates the initiation of a new national oral health survey in the coming years, this study establishes a critical historical baseline, providing the necessary evidence to inform the design of future strategies, and serves as a benchmark to evaluate the evolution of social inequalities in oral health over the last decade.

## 5. Conclusions

This study demonstrates a consistent association between dental caries and multidimensional poverty in Colombia, highlighting that oral health inequalities are driven by the accumulation of structural deprivations. Consequently, oral health should be considered a tangible indicator of structural inequality and included in social progress metrics. These findings support the design of comprehensive public health strategies that move beyond individual risk management to address the broader social determinants of health, particularly within the most vulnerable households.

## Figures and Tables

**Table 1:** Distribution of caries: individuals without caries, with caries in one tooth, and with caries in two or more teeth according to data on sociodemographic characteristics and caries prevalence per household. ENSAB IV (Spanish Acronym), 2014.

Characteristics of Household Members With and Without Dental Caries, n = 20.493	Total	Without Caries (67.1%)	Dental Caries in 1 Tooth (16.8%)	Dental Caries in 2 or More Teeth (16.1%)	*p*-Value
Age (mean) (SE)	22.32 (20.4)	18.70 (20.6)	28.10 (18.8)	31.40 (20.0)	0.000
Sex					
Female	56.10	66.11	17.69	16.20	0.006
Male	43.90	68.40	15.80	15.80	
Education (mean years) (SE)	7.13 (4.39)	7.88 (4.0)	8.32 (3.8)	7.24 (4.5)	0.000
Health System					
Contributive	34.50	72.33	15.44	12.23	0.000
Subsidized	54.60	63.36	17.51	19.13	
Exceptional	3.90	75.19	14.80	10.01	
Special	0.10	78.91	7.04	14.05	
Not insured	6.90	62.33	19.72	17.95	
Ethnic Group					
Indigenous	5.30	57.27	19.54	23.19	0.000
Mixed Ethnicity	41.30	65.78	17.79	16.43	
White	27.40	69.71	15.51	14.78	
Afro-Colombian	9.80	64.98	16.17	18.85	
Other	16.20	68.29	16.31	15.41	
Forced Displacement	13.40	60.66	19.38	19.96	0.000
**Characteristics of the Household** **,** **n = 14.964**		**No Members with Dental Caries**	**Members** **with Dental Caries**		***p*** **-Value**
Area					
Municipal Capital	80.50	60.34	39.66		0.000
Urban	8.10	52.16	47.84		
Rural	11.40	48.79	51.21		
Percentage, male-headed	67.80	58.30	41.70		0.111
Percentage, female-headed	32.20	59.00	41.00		
Head-of-household average age	45.60 (14.05)	45.90 (14.29)	45.30 (13.70)		0.000
Head-of-household education level (percentage)					
None	5.10	51.32	48.68		0.000
Primary	40.70	55.35	44.65		
Secondary	36.70	58.19	41.81		
Technical	8.60	65.17	34.83		
Undergraduate	5.90	65.03	34.97		
Postgraduate	1.50	73.41	26.59		
Region of Residence					
Atlantic	17.70	49.33	50.67		0.000
Asia	16.60	54.04	45.96		
Central	17.10	64.90	35.10		
Pacific	16.60	60.57	39.43		
Bogotá	16.10	62.69	37.31		
Orinoquia/Amazonía	15.90	55.39	44.61		

**Table 2:** Crude and adjusted regression models between MPI components and dental caries in multidimensionally poor households.

		Non-Adjusted Model				Adjusted Model			
Multidimensional Poverty Index		RR	*p* > |z|	[95% CI]		RR	*p* > |z|	[95% CI]	
Dimension	Indicator								
Educational conditions of the household	Low educational attainment	1.27	0.001	1.24	1.31	1.24	0.001	1.20	1.28
	Illiteracy	1.04	0.031	1.00	1.08	1.00	0.83	0.96	1.03
Conditions of childhood and youth	School non-attendance	1.09	0.004	1.03	1.16	1.03	0.35	0.97	1.09
	School lag/educational backwardness	1.12	0.001	1.09	1.15	1.03	0.01	1.01	1.06
	Child labor	1.16	0.001	1.10	1.22	1.10	0.001	1.04	1.15
Employment	Unemployment	1.04	0.007	1.01	1.07	1.03	0.04	1.0	1.06
Health	Health insurance coverage	1.09	0.001	1.06	1.12	1.08	0.001	1.05	1.11
	Access to oral health services given a need	0.85	0.001	0.83	0.88	0.84	0.001	0.81	0.86
Housing conditions and access to public services	Access to improved water source	1.09	0.001	1.04	1.14	1.02	0.48	0.97	1.07
	Housing conditions	1.19	0.001	1.13	1.24	1.12	0.001	1.07	1.18
	Overcrowding	1.08	0.001	1.05	1.11	1.05	0.001	1.02	1.08

**Table 3:** Incidence and intensity of the MPI at the national level and for households containing members with and without dental caries.

	Population %	Incidence %	IC		Intensity %	IC		Adjusted Model	IC	
National	100	55.7	55.3	56.0	46.1	46.0	46.2	0.256	0.255	0.258
Households containing members without dental caries	57.7	52.6	52.1	53.1	45.6	45.4	45.7	0.240	0.237	0.242
Households containing members with dental caries	42.3	59.9	59.3	60.5	46.7	46.5	46.9	0.280	0.277	0.283

**Table 4:** Association between presence of caries according to multidimensional poverty and other household conditions.

Caries in the Household	IRR	Std. Err.	z	*p* > |z|	[95% Conf. Interval]	
Multidimensional Poverty						
Not Poor	-	-	-	-	-	-
Poor	1.078	0.014	5.650	0.000	1.050	1.107
Sex of the head of household						
Male	-	-	-	-	-	-
Female	1.030	0.014	2.180	0.030	1.003	1.057
Head-of-household education level						
None	-	-	-	-	-	-
Primary	0.959	0.027	−1.520	0.129	0.908	1.012
Secondary	0.926	0.028	−2.580	0.010	0.873	0.982
Technical or higher	0.795	0.027	−6.770	0.000	0.744	0.849
Head-of-household ethnicity						
Indigenous	-	-	-	-	-	-
Mixed Ethnicity	0.931	0.024	−2.720	0.006	0.885	0.980
White	0.876	0.024	−4.860	0.000	0.830	0.924
Other	0.817	0.024	−7.030	0.000	0.772	0.864
Black-Afro	0.947	0.028	−1.820	0.069	0.893	1.004
Zone of the household						
Municipal Capital	-	-	-	-	-	-
Urban	1.112	0.025	4.690	0.000	1.064	1.163
Rural	1.167	0.024	7.650	0.000	1.122	1.214
Socioeconomic Stratum						
1	-	-	-	-	-	-
2	0.935	0.014	−4.410	0.000	0.908	0.963
3	0.874	0.019	−6.220	0.000	0.838	0.912
4	0.804	0.041	−4.270	0.000	0.727	0.888
5	0.841	0.019	−7.640	0.000	0.804	0.879
6	0.860	0.018	−7.190	0.000	0.826	0.896
Region						
Atlantic	-	-	-	-	-	-
Asia	0.937	0.020	−3.030	0.002	0.898	0.977
Central	0.707	0.016	−15.520	0.000	0.677	0.739
Pacific	0.761	0.016	−12.810	0.000	0.730	0.793
Bogotá	0.834	0.019	−7.940	0.000	0.797	0.872
Orinoquía/Amazonía	0.851	0.018	−7.660	0.000	0.817	0.887
Head-of-household average age	0.997	0.001	−6.030	0.000	0.996	0.998
_constant	0.655	0.032	−8.610	0.000	0.595	0.721

Socioeconomic status is an official classification that groups households according to their material conditions and urban environment, from stratum 1 (lowest) to stratum 6 (highest). This variable is a widely used proxy for measuring social and economic inequalities [19].

## Data Availability

Restrictions apply to the availability of these data. Data were obtained from Ministry of Health and Social Protection of Colombia and are available at [https://www.minsalud.gov.co/sites/rid/Lists/BibliotecaDigital/RIDE/VS/ED/GCFI/estudio-nacional-salud-bucal-ensab-iv.zip] (acceded on 19 February 2026). Access to the dataset requires a permission request to the Ministry via email at repositorio@minsalud.gov.co.

## Abbreviations

The following abbreviations are used in this manuscript:

ENSAB IV	IV National Oral Health Survey, Colombia
MPI	Multidimensional Poverty Index
AF	Alkire–Foster Method
WHO	World Health Organization
PSU	Primary Sampling Unit
SGSSS	General Social Security System for Health
DANE	Administrative Department of National Statistics
ENCV	National Quality of Life Survey

## Appendix A

**Table A1:** MPI DANE vs. MPI ENSAB IV indicators.

Dimension	Variable	Operational Definition	Cutoff Point	Question in ENCV	Question in ENSAB IV
Household education conditions	Educational achievement	This indicator is measured based on the average level of education of individuals aged 15 years and over within the household. However, it is worth noting that household members for whom preschool is the highest level of education, zero years of schooling is assigned. In terms of the cutoff point, a household is considered deprived when the average years of schooling of its members aged 15 and over is below nine [25]. However, when there are no household members aged 15 years and over, the household is automatically considered deprived in terms of educational achievement.	9 years	Average schooling of persons aged 15 and over in the household.	What is the highest level of education achieved by XXX? And what is the last grade or year passed at that level?
	Literacy	This indicator is defined as the percentage of people aged 15 or above in the household who know how to read and write. A household is considered deprived if at least one household member (i.e., less than 100%) aged 15 or older does not know how to read or write. When there are no household members aged 15 years old or over, the household is considered deprived.	100%	Percentage of persons in the household aged 15 and over who can read and write.	
Childhood and youth conditions	School Attendance	This indicator is calculated based on the proportion of school-age children (6 to 16 years old) in a household who attend an educational institution. According to this indicator, a household is considered deprived if at least one child (i.e., less than 100%) aged between 6 and 16 years old does not attend school. Households with no children between 6 and 16 years old are not considered deprived in this indicator.	100%	Percentage of children aged 6 to 16 in the household who attend school.	Where does XXX spend most of their time?
	No School lag	School lag is calculated for households with children between the ages of 7 and 17. The school lag of each child is defined as the difference between the number of legally expected years of schooling by age and the number of school years completed. The legally expected years of schooling by age are defined by the Sector Plan for Education 2006–2010 presented by the National Ministry of Education. A household is considered deprived in this variable if any child between 7 and 17 years is lagging in school. In other words, the desired result is that 100% of children in a household are without school lag. Households with no children between 7 and 17 years old are not deprived in this indicator.	100%	Percentage of children and youth (7–17 years old) in the household who are not behind in school (according to national standards).	What is the highest level of education achieved by XXX? And what is the last grade or year passed at that level?
	Access to child care services	This indicator assesses the percentage of children aged 0 to 5 years in each household who have simultaneous access to all childcare services (health, proper nutrition, and adult supervision or education). A household is considered to be deprived in access to childcare services if there is at least one child between 0 and 5 years old with no simultaneous access to all childcare services. Thus, a household is not deprived if its children under the age of 5 (i) spend most of the week at a community home, nursery or preschool, or are under the care of a responsible adult [26]; (ii) are covered by health insurance; and (iii) receive lunch in the care facility where they spend most of their time (a community home, nursery, or preschool).	100%	Percentage of children aged zero to five in the household with simultaneous access to health, nutrition, and early childhood education.	Not available
	Children not working	According to the International Labour Organization (ILO) [28] and the Colombian National statistical Department (DANE), child labor refers to children under 18 years old carrying out household chores for more than 15 h per week, children under 14 years old being classified as employed, and children under 18 years old being involved in hazardous work. Given the data constraints of the LSMS, the CMPI only includes the percentage of children in the household aged between 12 and 17 who are employed. The indicator of children not working is defined as the percentage of children who are not part of the labor market. A household is deprived in this variable if at least one child between 12 and 17 years old is employed. A household with no children between 12 and 17 years old is considered not deprived.	100%	Percentage of children aged 12 to 17 in the household who are not part of the labor market.	Do they work? And do they contribute money to the household?
Employment	Nobody in long-term unemployment	This indicator measures the percentage of the economically active population [30] (EAP) in the household that has been unemployed for more than 12 months.	100%	Percentage of the economically active population in the household who are not long-term unemployed (more than 12 months)	
	Formal employment			Percentage of the economically active population in the household who are employed and registered with a pension fund (proxy for informality)	Not available
Health	Health insurance	Health insurance coverage is defined as the proportion of household members covered by the Social Security Health System. A household is deprived if any of its members is not affiliated with a health insurance regime. Given that the access-to-childcare-services variable takes into account the health insurance status of children between 0 and 5 years old, this indicator is measured only for the population older than five.	100%	Percentage of household members over the age of five insured by Social Security Health Insurance	Which health system are they enrolled in?
	Access to health services	This indicator measures the proportion of people in a household who have access to health services when required. A household is not deprived in access to healthcare services if all of its members who, in the last thirty days, have experienced an illness, an accident, dental problems, or any other health issues that have not required hospitalization have been attended by a doctor, specialist, dentist, therapist or health institution. Households where nobody has had a need for healthcare services are not considered to be deprived.	100%	Percentage of household members who access institutional health services when faced with a pressing need.	For children: Have they ever made an appointment or taken XXX to the dentist and NOT been seen? For adults: Have they ever been denied dental care in the last 12 months?
Access to public utilities and adequate housing conditions	Access to water source	This indicator was defined using WHO-UNICEF guidelines [33], where urban households are considered deprived when they have no access to public water services. In rural areas, households are considered deprived when they have no access to public water services and the water used to prepare food is obtained from a well, rainwater, a river, a spring water source, a public tap or standpipe, a water truck, a water carrier, or any source other than piped water.	1	Urban household: considered private if it does not have public water supply service in the home. Rural household: considered private when water for food preparation is obtained from a well without a pump, from rainwater, a river, a spring, a tanker truck, a water carrier, or another source.	Is their home supplied with water through…? Differences between urban and rural households are taken into account for deprivation.
	Adequate elimination of sewer waste	In this indicator, urban households without access to a public sewer system are considered deprived. Rural households are considered deprived if they have a toilet without a sewer connection or a latrine, or if they simply do not have a toilet.	1	Urban household: considered private if it does not have public sewerage services. Rural household: considered private if it has an unconnected toilet, low tide, or no sanitation services.	Not available
	Adequate floors	Households with dirt floors are considered deprived.	1	Households with dirt floors are considered deprived.	For these two variables, the following question was used: What type of housing do you live in? Respondents were classified as non-private if they lived in an apartment, house, or rural ranch, and private if they lived in makeshift housing, an urban ranch, collective housing, or another type of housing.
	Adequate external walls	An urban household is considered deprived when the exterior walls are built of untreated wood, boards, planks, guadua or other vegetation, zinc, cloth, cardboard, or waste material, or when no exterior walls exist. A rural household is considered deprived when exterior walls are built of guadua or other vegetation, zinc, cloth, cardboard, or waste materials, or if no exterior walls exist.	1	Urban household: considered private if the exterior walls are made of rough wood, boards, planks, bamboo, other plant materials, zinc, fabric, cardboard, debris, or have no walls. Rural household: considered private if the exterior walls are made of bamboo, other plant materials, zinc, fabric, cardboard, debris, or have no walls.	
	No critical overcrowding	This indicator considers the number of people sleeping per room, excluding the kitchen, bathroom and garage	Urban: 3 or more people per room Rural: More than 3 people per room	Number of people per bedroom excluding kitchen, bathroom, and garage, and including living room and dining room	How many people live in this household? And how many rooms are used in this household as bedrooms (not counting bathrooms and kitchens)?
